# Effect of Different Protocols of Fixed-Time Artificial Insemination on Mucus, Ovarian Size, and Pregnancy of Mixed-Breed Cows in the Humid Tropics of Peru

**DOI:** 10.1155/2023/9942021

**Published:** 2023-05-18

**Authors:** Clavel Diaz-Quevedo, Alonso Ramírez García, Lenin Torres Bernal, Jorge Cáceres Coral, Gustavo Ampuero Trigoso, José Américo Saucedo-Uriarte

**Affiliations:** ^1^Estación Experimental Agraria San Ramón, Instituto Nacional de Innovación Agraria, Yurimaguas 16501, Peru; ^2^Facultad de Zootecnia, Agronomía Ciencias Biológicas y Acuicultura, Universidad Nacional Autónoma de Alto Amazonas, Yurimaguas 16501, Peru; ^3^Facultad de Medicina Veterinaria, Universidad Nacional de San Martín, Tarapoto 22201, Peru; ^4^Dirección de Desarrollo Tecnológico Agrario, Instituto Nacional de Innovación Agraria, Lima 15024, Peru; ^5^Estación Experimental Agraria El Porvenir, Instituto Nacional de Innovación Agraria, Juan Guerra 22400, Peru

## Abstract

The objective of the research was to evaluate three synchronization protocols for fixed-time artificial insemination (FTAI) in *Bos indicus* with *Bos taurus* crossbred cows. Forty-five cows of 5 ± 1.6 years were selected. The Jsynch treatment consisted of the application of an intravaginal device plus benzoate; on day 8, the device was removed and PGF2*α*, estradiol cypionate plus eCG was applied, and the FTAI was performed at 48–52 hours after. Conventional treatment consisted of the application of an intravaginal device plus benzoate; on day 8, the device was removed and PGF2*α* plus eCG was injected, and on day 9, estradiol benzoate was administered, and FTAI was performed at 30 hours. The Ovsynch treatment consisted of the application of GnRH, and on day 8, PGF2*α* was applied, on day 9, GnRH was applied again, and on day 10, FTAI plus GnRH was applied. The diagnosis of pregnancy was determined by transrectal ultrasound 45 days after the FTAI. The Jsynch (39.1%) and conventional (39.1%) treatment showed higher pregnancy compared to the Ovsynch treatment (21.7%) of pregnancy. The presence of crystalline mucus at the time of the FTAI was significantly associated with pregnancy. The results of this investigation indicate that it is possible to obtain acceptable pregnancy rates with the FTAI in *B. indicus* with *B. taurus* crossbred milk-producing cows, and treatments with progesterone-releasing devices plus eCG can improve the reproductive performance of cows.

## 1. Introduction

In tropical and subtropical regions of the world, the handling of zebu cattle (*B. indicus*) predominates, attributed to its adaptive and productive characteristics [1, 2]. However, bovine production in the tropics is generally low, due to nutritional, environmental, genotype, and inadequate reproductive handling practices [3], reflected in the reduction of fertility in beef and dairy cows, which affects ovarian function, estrus expression, oocyte health, and embryonic development [4–7]. In this context, an alternative to overcome these inefficiencies has been developed, biotechnologies such as artificial insemination and fixed-time artificial insemination (FTAI) [8], with the use of estrus synchronization protocols through hormonal treatments that allow the genetic improvement [9]. Estrus synchronization is a key tool when using artificial insemination to reduce the time and labour required to detect estrus, bearing in mind that a successful protocol requires precise control of follicular development and regression of the corpus luteum [10].

In the tropical regions of Peru, genetic improvement with artificial insemination is still a limitation and the studies carried out are scarce. Therefore, it is necessary to carry out research work regarding the use of mechanisms that help improve the reproductive aspects of cattle in these tropical areas. An alternative is to use estrus synchronization protocols for FTAI to ensure that livestock production exceeds profitability and efficiency standards that allow economic reactivation in this part of the country. In this sense, we evaluated the effect of three synchronization protocols for fixed-time insemination on the pregnancy of *Bos indicus* and *Bos taurus* crossbred cows in the humid tropics of Peru.

## 2. Materials and Methods

### 2.1. Study Area and Population

The experiment was conducted from May to August 2022. A total of 45 crossbred females were selected and worked in four cattle herds in the province of Alto Amazonas (latitude 5° 53′ 38″ south and longitude 76° 6′ 25″ west), with an average annual temperature from 21°C to 32°C, relative humidity from 74.5% to 81.5%, and at 107 masl. For the selection of the animals, they underwent a gynaecological clinical examination with a 7.5 MHz linear frequency transducer (Draminski iScan Ultrasound Scanner 2020, Poland). The 45 animals that were selected did not present abnormalities in the reproductive tract. The animals were fed in an extensive system, grazed on *Brachiaria brizantha* grass, with mineral salt supplementation, and provided access to water *ad libitum*. The cows had an average age of 5 ± 1.6 years and were divided into three groups. Body condition was rated with a score between 2.75 and 3.50 (scale 1–5), an evaluation carried out before the experiment [11]. The data were evaluated according to treatment (conventional, Jsynch, and Ovsynch), breed (Gyr with Brahman, Gyr with Holstein, Browns Swiss with Brahman, and Brown Swiss with Holstein), number of calvings (0 calving, 1–3 calving, and 4 to more calving), ovary size (small is 1.0–2.4 cm, normal is 2.5–3.5 cm, and big is 3.6–5.5 cm), mucus at the time of insemination (without mucus, with crystalline mucus, with crystalline mucus and white spots, and with yellowish mucus), and body condition (2.75 and 3.00).

**Figure 1:**
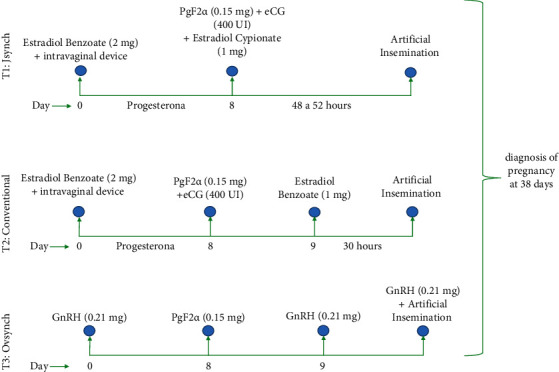
Description of the protocols.

All cattle were fed following the feeding routine in the same cattle herd. Cows were synchronized using the three groups. T1: Jsynch, day 0 it was 2 ml of estradiol benzoate was applied (2 mg) plus a progesterone intravaginal device (1.2 g), on day 8 device withdrawal and 0.15 mg of PgF2*α* plus 400 IU of eCG, and 1 mg of estradiol cypionate and artificial insemination was performed 48–52 hours after. T2: conventional, on day 0 it was 2 ml (2 mg) of estradiol benzoate was applied plus a progesterone intravaginal device (1.2 g), on day 8 the device was removed, 0.15 mg PgF2*α* plus 400 IU of eCG was injected, and on day 9 it was 1 mg of estradiol benzoate administered, and artificial insemination was performed 30 hours after. T3: Ovsynch [12]. on day 0 it was 5 ml (0.21 mg) of GnRH was applied, on day 8 it was 2 ml (0.15 mg) of PgF2*α* was applied, on day 9 it was 5 ml (0.21 mg) of GnRH was applied, and day 10 it was 5 ml (0.21 mg) GnRH was applied and artificial insemination was performed (Figure 1). Pregnancy diagnosis was performed with ultrasonography (45 days after insemination) with a 7.5 MHz linear frequency transducer (Draminski iScan Ultrasound Scanner 2020, Poland). A positive pregnancy was observed in the uterine horn and the presence of amniotic fluid with a black tone (anechogenic) that surrounds the embryo with a white tone (echogenic), and for a negative gestation, the uterine horn was observed empty in rounded ways.

The cost of each protocol for estrus synchronization was determined by analyzing the costs per animal treated and globally for the entire sample for each protocol group.

### 2.2. Data Analysis

For the processing of the results obtained, descriptive statistics were used for parameter estimation and the chi-squared test via goodness of fit for the comparison of the categories and independence tests to determine the relationship between the percentage of pregnancy in relation to the treatment, breed, number of calvings, ovary size, mucus at the time of insemination, and body condition. In addition, the percentage of pregnancy in relation to the treatment according to ovary size and body condition was also determined. All the analyses were processed in the SPSS v 26 software.

## 3. Results and Discussion

**Figure 2:**
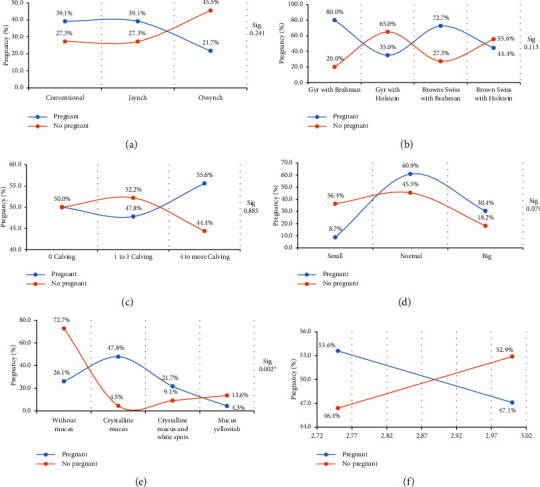
Percentage of pregnancy in cows. Pregnancy according to (a) treatment, (b) cross breed, (c) calving number, (d) ovary size, (e) presence of cervical mucus, and (f) body condition.

The average values of the percentage of pregnancy in relation to the treatment, breed, calving number, ovary size, mucus at the time of insemination, and body condition are given in Figure 2. No significant association was found using the chi-squared test (*p* > 0.05) between treatments (Figure 2(a)). With the application of the conventional protocol and Jsynch, 39.1% pregnancy was obtained (8/15 cows), while with the Ovsynch protocol, 21.7% pregnancy was registered (5/15 cows). By applying the Jsynch and conventional protocols in cows of the zebu breed group and their crosses, it has been identified that at 30 days of diagnosis, the conventional protocol presents the same percentage of pregnancy compared to the Jsynch [13]. These scientific reports support our findings because they indicate that applying the Jsynch protocol promotes a longer proestrus, managing to make the uterus environment more receptive with higher progesterone levels and a better developed corpus luteum, helping better implantation and maintenance of pregnancy [14, 15]. In this sense, our results obtained after the application of the Jsynch protocol and the conventional one favour its use in the tropical conditions of eastern Peru.

No significant association (*p* > 0.05) was found between the racial group with the percentage of pregnancy (Figure 2(b)). The highest percentage of pregnancy was the crossbreed of Gyr with Brahman, followed by the Browns Swiss crossbreed with Brahman, obtaining 80% and 72% of pregnancy, respectively. The groups of the other breeds showed a pregnancy rate of less than 45%. In Braford heifers (crossing *B. taurus* with *B. indicus*), when applying the Jsynch protocol associated with eCG, it presented a 60.4% pregnancy rate [16]. In Holstein–Friesian crossed cows with Gyr, when applying a conventional protocol, a 72.7% pregnancy percentage was reported [17]. In cows of the Holstein breed, pregnancy percentages of 34.7%–48.7% were observed with the Ovsynch protocol, 48.0%–67.8% with the Ovsynch plus P4 protocol, and 50.0%–72.4% with the Jsynch protocol [18].

The results show that the cows that have more than four calving, followed by the nulliparous, showed a better pregnancy percentage of 55% and 50%, respectively. Cows from 1 to 3 calving showed 47% pregnancy, being the lowest (Figure 2(c)). In pubertal heifers of the Nelore breed, applying the Jsynch protocol, they presented a percentage of 50.8 of pregnancy [19]. In first-time heifers, the Jsynch protocol can be successfully applied because it is observed that they have an adequate response to pregnancy [20]. The cows that presented ovaries of normal size reported a higher percentage of pregnancy compared to the other groups (*p* > 0.05) (Figure 2(d)).

A significant association (*p* < 0.05) was found between the percentage of pregnancy and cervical mucus (Figure 2(e)). 47.8% of pregnancy was observed when they present clean and crystalline mucus; otherwise, it happened in cows that presented dirty mucus (white and yellow spots) reducing the percentage of pregnancy to 26% and 4%, respectively. The presence of cervical mucus at the time of the FTAI is due to the increase in estrogen, a crystalline mucus is related to a greater number of pregnant cows [21]. In addition, our findings agree with the reports of [22], which indicate that the presence of crystalline mucus in *B. indicus* cows before the FTAI is a sign of estrus [21]. This same effect was also seen in *B. indicus* cattle and their crosses; cows that presented fluid in estrus and crystalline mucus at the time of the FTAI presented a higher percentage of pregnancy compared to cows that did not present such signs [23, 24]. This is explained because the cervical fluid fulfils the function of transport and survival of the spermatozoa on the way to the encounter with the ovum [25, 26]. The absence, limited mucus, or yellowish mucus can affect the functionality of the female reproductive tract because poor-quality cervical mucus can be caused by the presence of a poor-quality follicle [27–29]. Likewise, cows with absence or crossbreed noncrystalline cervical mucus may affect biocommunication that is based on pheromones present in the cervical mucus and therefore would affect sexual biostimulation and the presence of estrus [30–32].

A higher pregnancy percentage of more than 53% was found in cows that presented a body condition of 2.75 and a lower percentage of 47% in cows that presented a body condition of 3.00 (Figure 2(f)). Similar results are observed in the studies by Yánez-Avalos et al. [33], who evaluated 448 multiparous cows of Brown Swiss breeds and their crosses with *B. indicus* with body conditions of 2.50–3.00, obtaining a 51.3% pregnancy and 48.6% empty cows. In addition, of 120 crossbred zebu cows with a body condition of 2.50, 57.5% pregnancy was obtained by applying GnRH at different stages of the estrus synchronization protocol [34].

The percentage of pregnancy per treatment according to ovary size of crossbred cows is not associated with ovary size (*p* > 0.05) (Table 1). Cows with small ovaries presented 50% pregnancy for the conventional and Ovsynch treatment and 0% pregnancy for the Jsynch protocol. Otherwise, it was reported when the cow presents an ovary of normal size with respect to the percentage of pregnancy, the Jsynch protocol presented 50% pregnancy, followed by the conventional with 35.7% and 14.3% for the Ovsynch protocol. What differs with the results obtained when the cows have large ovaries, where the highest percentage of pregnancy was recorded using the conventional protocol with 42.9% followed by 28.6% pregnancy for the Jsynch and Ovsynch protocols. When applying an Ovsynch protocol in Holstein–Gyr crossbred cows, at 30 days of insemination, a pregnancy percentage of 39% was obtained, and at 60 days, it was reduced to 34.8% [35]. Our results are inferior to the results reported by Bottino et al. [35], possibly because they preprogrammed synchronization through induction of persistent dominant follicles using a progesterone device prior to the Ovsynch protocol that produced double Ovsynch-like patterns of follicular growth, regression, and fertility [35].

**Figure 3:**
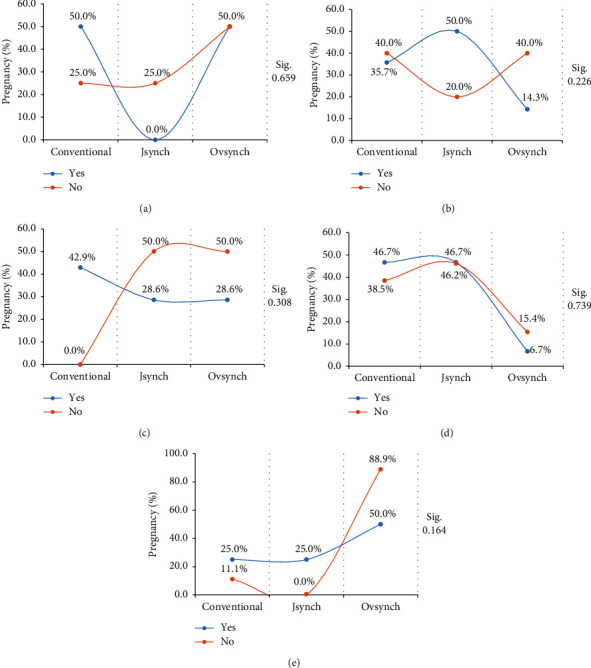
Pregnancy (%) in relation to treatment according to ovary size and body condition: (a) according to small ovary, (b) according to normal ovary, (c) according to big ovary, (d) according to body condition of 2.75, and (e) according to body condition of 3.

The pregnancy expressed as a percentage and in relation to the treatment according to ovary size and body condition is shown in Figure 3. In our research, we found no significant association between treatments according to small ovary (Figure 3(a)), according to normal ovary (Figure 3(b)), and according to body condition of 3 (Figure 3(e)). Our results are consistent with previous studies suggesting a trend for the Jsynch protocol that resulted in a higher pregnancy rate in heifers compared to the conventional protocol [36]. However, a different pregnancy response was observed when the cows showed big ovaries, where the highest percentage of pregnancy was recorded with the Ovsynch protocol with 50% and 25% for the other protocols (Figure 3(c)). According to results obtained for the body condition of 2.75 in cows, the highest percentage of pregnancy was recorded when using the Jsynch and conventional protocols with 46.7% compared to Ovsynch (*p* > 0.05) (Figure 3(d)). Studies such as that of Mion et al. [37] indicate that there is an association of the Jsynch protocol with the detection of heat and the anticipation of the moment of insemination and that it could increase the percentage of pregnancy by avoiding asynchrony between insemination and ovulation.

The purpose of the estrus synchronization protocols for FTAI is to increase reproductive rates through the use of hormones at a moderate cost to obtain greater economic benefit. The application of the conventional protocol had a cost of 262.8 USD per group and 17.5 USD per cow. In the Jsynch protocol, there was a cost of 269.5 USD per group and 18.0 USD per cow, and in the Ovsynch protocol, there was a cost of 212.6 USD per lot and 14.2 USD per cow (Table 2). Rodríguez–Irazoqui et al. [38] indicated that when applying the Ovsynch protocol, the cost of hormonal treatments was USD 33. The analysis of the costs for each hormonal treatment is of vital importance to have economic efficiency, in this sense, to analyze the shortening of the service period and to concentrate the calving's and groups of homogeneous calves [38, 39]. In our study, there were no differences between treatments; however, treatment with GnRH was less costly. Our results are consistent with the study of Fricke et al. [40] that indicated a GnRH treatment used for ovulation synchronization, and timed AI in lactating dairy cows reduces synchronization costs per cow and per pregnancy without compromising the efficacy of the synchronization protocol.

## 4. Conclusions

In *B. indicus* with *B. taurus* crossbred cows, the Jsynch and conventional treatment produced a better pregnancy rate than the Ovsynch treatment. The crystalline cervical mucus is associated with the pregnancy of crossbred cows and presents a higher pregnancy rate. The cost for each one of the FTAI protocols is more accessible to the economy of the cattle producer than buying a bull of high genetic quality and giving it adequate management that expresses its genetics. It is possible to obtain acceptable pregnancy rates with fixed-time insemination in *B. indicus* with *B. taurus* crossbred cows and thus obviate the inconvenience of estrus detection.

## Figures and Tables

**Table 1:** Pregnancy according to the number of calving and ovary size.

Id	Calving			
	0 calving (%)	1–3 calving (%)	4 to more calving (%)	Sig
*Ovary size*				
Small	10.0	50.0	40.0	0.679
Normal	12.5	54.2	33.3	
Big	0.0	45.5	54.5	

*Cervical mucus*				
Without mucus	13.6	45.5	40.9	0.723
Crystalline mucus	8.3	41.7	50.0	
Crystalline mucus and white spots	0.0	71.4	28.6	
Mucus yellowish	0.0	75.0	25.0	

*Body condition*				
2.75	7.1	53.6	39.3	0.839
3.00	11.8	47.1	41.2	

**Table 2:** Cost recorded for each protocol.

Treatments	Units	Cows (N)	Cost (USD)	Dose	Price/animal (USD)	Price/group (USD)
*Conventional*						
Intravaginal device	10 device pack	15	91.0	1	9.1	136.3
Estradiol benzoate	100 ml bottle		26.0	2 mg	0.5	7.8
PGF2*α*	20 ml bottle		24.7	0.15 mg	2.5	37.0
eCG	25 ml bottle		62.4	1 mg	20.0	77.9
Estradiol benzoate	100 ml bottle		26.0	1 mg	5.2	3.9
Subtotal (USD)					17.5	262.8

*Jsynch*						
Intravaginal device	10 device pack	15	91.0	1	9.1	136.5
Estradiol benzoate	100 ml bottle		26.0	2 mg	0.5	7.8
PGF2*α*	20 ml bottle		24.7	0.15 mg	2.5	37.1
Estradiol cypionate	50 ml bottle		33.8	1 mg	0.7	10.1
eCG	25 ml bottle		62.4	1 mg	5.2	78.0
Subtotal (USD)					18.0	269.5

*Ovsynch*						
GnRH	50 ml bottle	15	39.0	0.21 mg	3.9	58.5
PGF2*α*	20 ml bottle		24.7	0.15 mg	2.5	37.1
GnRH	50 ml bottle		39.0	0.21 mg	3.9	58.5
GnRH	50 ml bottle		39.0	0.21 mg	3.9	58.5
Subtotal (USD)					14.2	212.6

## Data Availability

All data pertaining to the current study are available from the corresponding author upon a reasonable request.
